# Dynamic dopamine signaling, prediction error, and microcircuit mechanisms in stress-induced depression-like behaviors

**DOI:** 10.3389/fcell.2026.1948686

**Published:** 2026-10-12

**Authors:** Ashraf Mahmud, Ljupcho Efremov

**Affiliations:** Liberal Arts Department, American University of the Middle East, Egaila, Kuwait

**Keywords:** chronic stress, depression, depression-like behaviors, dopamine, prediction error, social defeat, ventral tegmental area

## Abstract

Chronic stress contributes to major depressive disorder (MDD) phenotypes by disrupting coordinated neuromodulatory systems and their corresponding computational frameworks. In this review, we explore how the dynamic interplay of ventral tegmental area dopamine (DA) signaling, microcircuit gating, and prediction error mechanics orchestrates stress-induced depressive behaviors, such as anhedonia and amotivation. Rather than acting as the sole driver of depression, pathway-specific neuroadaptations across mesolimbic ( VTA→NAc ) and mesocortical ( VTA→PFC ) dopaminergic projections operate within a broader multi-transmitter landscape, interacting with serotonergic, noradrenergic, cholinergic, and glutamatergic/GABAergic systems. These signaling anomalies often translate into computational deficits in reinforcement learning, manifesting as blunted striatal reward prediction error (RPE) signals that scale directly with anhedonia severity. Chronic stress also disrupts cortical microcircuit gating governed by dopamine $D_{1}$ and $D_{2}$ receptors, inducing cellular senescence and collapsing ensemble synchrony within the prefrontal cortex and interconnected networks. Furthermore, specialized subcortical loops, including inputs from the nucleus accumbens (NAc), ventral hippocampus (vHIPP), lateral habenula (LHb), and locus coeruleus (LC), recalibrate overall susceptibility versus resilience by altering DA firing dynamics and population activity pools, often in a sex-specific manner. By bridging synaptic dopamine dynamics, subcortical prediction errors, and cortical ensemble synchrony, we propose a unified, multi-level framework demonstrating how stress-induced depressive behaviors result from a progressive breakdown in the brain’s predictive coding and adaptive coping networks.

## Introduction

Major depressive disorder (MDD) is a highly disabling, clinically heterogeneous multifactorial psychiatric condition. While the etiology of MDD is multifaceted and involves a complex interplay of genetic, neuroendocrine, and environmental factors, chronic stress remains a primary predictor for the onset, maintenance, and symptomatic exacerbation of depressive episodes (Ormel et al., 2001; Tennant, 2002). At the macro-circuit level, chronic exposure to psychological and physical stressors disrupts prefrontal cortex (PFC) networks and interconnected limbic structures, particularly those governing reward evaluation and motivational drive. Rather than causing uniform synaptic loss, chronic stress induces region- and cell-type-specific structural reorganization across frontostriatal and limbic brain regions, bringing diminished myelination, reduced regional gray-matter volume, dendritic arbor contraction, loss of dendritic spine density, and impaired synaptic plasticity in prefrontal and hippocampal pyramidal neurons, while paradoxically triggering dendritic hypertrophy and spinogenesis in subcortical structures such as the basolateral amygdala (BLA) and nucleus accumbens (NAc) (Fischer et al., 2016; Belleau et al., 2018; Hare and Duman, 2020; Mahmud et al., 2023; Mahmud and Karaman, 2026). Historically, the neurobiological framework of MDD were dominated by the monoamine hypothesis, largely focusing on serotonergic and noradrenergic deficits (Coppen, 1967; Schildkraut, 1967; Hirschfeld, 2000). However, the limited efficacy of classical monoaminergic reuptake inhibitors in treating core MDD symptoms, the frequent clinical refractoriness, particularly anhedonia, amotivation, psychomotor slowing, cognitive inflexibility, and deficits in learning, has catalyzed a paradigm shift toward the midbrain dopaminergic (DA) system. Dopamine signaling acts as the primary substrate for reward processing and learning, effort-based decision-making, reward prediction error (RPE) encoding, and cognitive control, and resolving how chronic stress destabilizes midbrain DA architecture is essential for understanding the core pathophysiology of depression.

Accumulating evidence suggests that stress-induced dopaminergic dysfunction cannot be conceptualized as a uniform or simple global deficit in DA transmission. Rather, chronic stress triggers highly divergent, complex, circuit-specific neuroadaptations that alter the foundational properties and mechanics of DA signaling. Although emerging studies indicate that dopamine neurons within the substantia nigra pars compacta (SNc) also contribute to affective and motor-motivational deficits and thus involved in depression-like behaviors (Winter et al., 2007; Yao et al., 2021), this review primarily focuses on the mesocorticolimbic dopamine system, originating in the ventral tegmental area (VTA), which is highly sensitive to environmental stressors. Under non-pathological conditions, the precise balance between slow, pacemaking tonic activity and rapid, burst-firing phasic events within VTA DA neurons encodes motivational salience and updates reinforcement learning parameters. Prolonged exposure to stress, however, disrupts these intrinsic firing dynamics, leading to aberrant DA release dynamics, often forcing a critical divergence between single-unit firing rates and the total pool of spontaneously active pacemaking neurons (Lammel et al., 2014; Grace, 2016). Crucially, the cellular consequences of this disruption are highly segregated by projection target. In the mesolimbic pathway projecting to the NAc, stress initiates maladaptive synaptic plasticity and alters $D_{1}$ vs. $D_{2}$ receptor trafficking, and disrupts hyperpolarization-activated cation currents ( $I_{h}$ ), directly underpinning anhedonic and amotivational phenotypes (Arnsten, 2015; Hare and Duman, 2020). Concurrently, in the mesocortical projections to the PFC, stress-induced DAergic dysregulation impairs local inhibitory microcircuits, induces dendritic spine retraction, and impairs $D_{1}R/D_{2}R$ -mediated signaling, manifesting as executive dysfunction and loss of top-down emotional regulation (Shinohara et al., 2018).

In this review, we discuss recent advances detailing how chronic stress-induced mechanisms exacerbate dopamine-mediated circuit reorganization to induce depression-like behaviors in rodent models and elevate MDD symptom severity in human. Particularly, we examine how stress alters VTA microcircuits, local neurotransmitter receptor dynamics, and RPE computations to initiate and sustain depressive phenotypes. Finally, we synthesize emerging insights across VTA sub-nodes, including reciprocal loops with the PFC, NAc, ventral hippocampus (vHIPP), lateral habenula (LHb), and locus coeruleus (LC) to elucidate stress vulnerability and resilience, highlighting novel targets for circuit-specific therapeutic interventions.

### Dopamine disruptions in MDD in humans

In humans, the core symptom of depression, anhedonia, is strongly linked to dysfunction within the brain’s reward system and, in particular, the mesolimbic DA system (Heshmati and Russo, 2015; Rincón-Cortés and Grace, 2020). Early clinical evidence indicated that the nigrostriatal DA system is underactive in depressed individuals (see review (Willner, 1983)). Subsequent work demonstrated that patients exhibit significant deficits in central norepinephrine and dopamine turnover, accompanied by a metabolic shift toward non-glucose energy sources, where overall clinical severity paradoxically correlates most strongly with brain dopamine turnover (Lambert et al., 2000). Furthermore, positron emission tomography (PET) imaging in depressed patients with prominent anhedonia reveals significantly lower dopamine transporter (DAT) binding compared to healthy controls, suggesting a compensatory downregulation driven by sustained low synaptic DA concentrations (Sarchiapone et al., 2006). Supporting this causal link, administration of α -methyl-p-tyrosine, a potent inhibitor of the rate-limiting dopamine biosynthetic enzyme tyrosine hydroxylase, induces or exacerbates depressive symptoms in both actively depressed and remitted individuals (Hasler et al., 2008). Expanding on these findings, a recent meta-analysis showed that while individuals with MDD show no baseline differences in striatal $D_{2}/D_{3}$ receptor availability or overall DA synthesis capacity, they exhibit significantly reduced striatal DAT availability (when evaluated with selective tracers) alongside lower striatal $D_{2}$ receptor availability (Mizuno et al., 2023). Together, these findings provide a nuanced molecular framework that helps reconcile clinical presentation with preclinical models.

Aligning with the Research Domain Criteria (RDoC) framework, dopaminergic dysfunction in humans selectively maps onto Positive Valence Systems, specifically governing hedonic responsiveness, effort-based cost-benefit computation, and psychomotor drive (Treadway and Zald, 2011; Barch et al., 2015). Conversely, core clinical features of MDD such as prolonged depressed mood/sadness, rumination, feelings of worthlessness/guilt, and suicidal ideation may reflect non-dopaminergic circuit disruptions, including Default Mode Network (DMN) hyper-connectivity, altered serotonergic and noradrenergic signaling, and prefrontal-amygdala E/I excitation/inhibition (E/I) imbalance (Menon, 2011). Recognizing this domain specificity reinforces the idea that dopamine circuit disruptions or adaptations may represent a symptom-specific endophenotype rather than an all-encompassing etiology for MDD.

### Physiology and heterogeneity of dopamine neurons

Emerging evidence highlights that midbrain dopaminergic neurons are highly heterogeneous, defined by distinct embryological ontogenies, unique transcriptomic profiles, and segregated circuit architectures that projection-specifically modulate discrete behavioral and physiological states (see reviews (Morales and Margolis, 2017; Garritsen et al., 2023; Peña et al., 2025)). Within VTA, DA neurons display highly organized tonic and phasic electrophysiological activities governed by multi-tiered regulatory circuits (Floresco et al., 2003; Paladini and Roeper, 2014). Their baseline pacemaker firing is primarily governed by the hyperpolarization-activated cation current (*I*_h_). Within the local intra-VTA microcircuit, tonic activity is tightly modulated by inhibitory checkpoints, including local GABAergic interneurons and D_2_ autoreceptor negative feedback loops.

Beyond local microcircuit control, VTA DA dynamics are shaped by a diverse array of extrinsic inputs. Excitatory glutamatergic inputs originate from the mPFC, the LHb, the laterodorsal tegmentum (LDTg), the bed nucleus of the stria terminalis (BNST), and the pedunculopontine nucleus (PPN) (Oishi and Lazarus, 2017). Notably, these glutamatergic afferents display striking projection-specific functional divergence: for instance, optogenetic phasic stimulation of LDT neurons projecting to the VTA induces conditioned place preference whereas stimulation of LHb-to-VTA projections induces conditioned place aversion (Lammel et al., 2012). Inhibitory control is similarly distributed, with GABAergic inputs arriving from the ventral pallidum (VP), the lateral hypothalamus (LH), the periaqueductal gray (PAG), dorsal raphe (DR), local intra-VTA GABAergic interneurons, and the tail of the VTA (tVTA), also known as the rostromedial tegmental nucleus (RMTg) (Omelchenko and Sesack, 2009; Oishi and Lazarus, 2017). Finally, this balance is further tuned by modulatory cholinergic inputs from the LDTg, noradrenergic signals from the LC and direct serotonergic inputs from the DR, which also innervate local VTA GABA interneurons to indirectly disinhibit or inhibit DA firing (see Figure 1) (Beier et al., 2015; Oishi and Lazarus, 2017).

**FIGURE 1:**
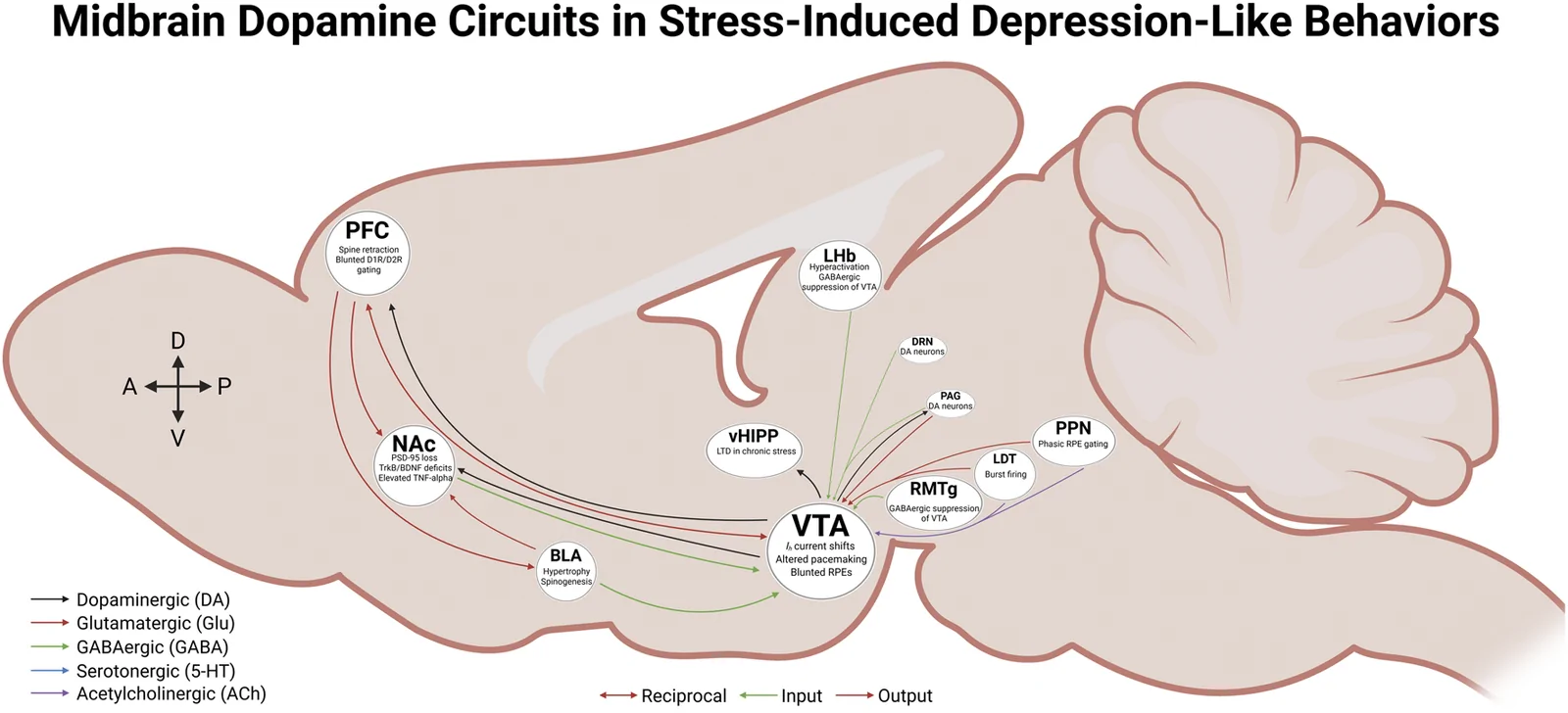
Midbrain dopamine circuits in stress-induced depression-like behaviors. Schematic representation of midbrain ventral tegmental area (VTA) dopamine (DA) projections to primary frontostriatal and limbic targets. Mesolimbic DA projections originating in the ventral VTA innervate medium spiny neurons (MSNs) within the nucleus accumbens (NAc) core and shell to regulate reward prediction error (RPE) computations, incentive salience, and effort-based cost-benefit valuation. Mesocortical projections from the dorsomedial VTA target pyramidal neurons and fast-spiking interneurons within the medial prefrontal cortex (mPFC), subserving top-down executive control and cognitive flexibility. Upstream subcortical structures dynamically shape VTA DA population firing activity and pacemaking states. Mesopontine inputs from the pedunculopontine nucleus (PPN) deliver top-down glutamatergic and cholinergic signals that gate millisecond-scale phasic RPE transients via local VTA nicotinic and muscarinic acetylcholine receptors, whereas the laterodorsal tegmentum (LDT) preferentially drives sustained burst firing and reinforcement. The lateral habenula (LHb) engages the rostromedial tegmental nucleus (RMTg) to deliver GABAergic inhibition onto VTA DA neurons during aversive or non-rewarding events. Basolateral amygdala (BLA) projections modulate local terminal DA release. Chronic stress exposure triggers highly divergent, projection-specific adaptations across mesocorticolimbic hubs. In the mPFC, stress drives apical dendritic arbor contraction and spine retraction on pyramidal neurons, destabilizing $D_{1}R/D_{2}R$ -mediated microcircuit gating. Conversely, BLA neurons undergo paradoxical dendritic hypertrophy and spinogenesis. Within the NAc, chronic stress downregulates postsynaptic density protein 95 (PSD-95) scaffolding, disrupting BDNF/TrkB trophic cascades while elevating TNF- α -mediated neuroinflammation. Presynaptically, altered dopamine transporter (DAT) clearance kinetics, membrane redistribution, and homeostatic shifts in hyperpolarization-activated cation currents ( $I_{h}$ ) blunts sub-second phasic DA transients, impairing RPE encoding and promoting anhedonic phenotypes. Abbreviations: BLA, basolateral amygdala; DA, dopamine; DAT, dopamine transporter; $D_{1}R/D_{2}R$ , dopamine receptor subtypes 1 and 2; $I_{h}$ , hyperpolarization-activated cation current; LDT, laterodorsal tegmentum; LHb, lateral habenula; NAc, nucleus accumbens; PFC, prefrontal cortex; PPN, pedunculopontine nucleus; PSD-95, postsynaptic density protein 95; RMTg, rostromedial tegmental nucleus; RPE, reward prediction error; TNF- α , tumor necrosis factor-alpha; TrkB, tropomyosin receptor kinase B; vHIPP, ventral hippocampus; VTA, ventral tegmental area.

Other than classical dopaminergic markers such as tyrosine hydroxylase (*Th*; the rate-limiting enzyme in dopamine biosynthesis) and the dopamine transporter (*Slc6a3*), a broader palette of homeodomain proteins and transcription factors, including *Nr4a2* (Nurr1), *Pitx3*, *Aldh1a1*, *Lmx1b*, and *Wnt1*, is used to define midbrain dopaminergic (mDA) lineages (Prakash and Wurst, 2006). Integrating these initial developmental markers with high-resolution transcriptomic profiling, Poulin and colleagues stratified mDA neurons into six distinct classifications defined by their developmental lineages, anatomical distribution, and projection targets: Cluster a: *Sox6* ^+^/*Aldh1a1* ^+^ neurons located primarily in the SN that project to the dorsolateral striatum; Cluster b: *Sox6* ^+^/*Aldh1a1* ^-^ neurons distributed across the SN and VTA that project to the dorsal striatum and NAc core; Cluster c: *Slc17a6* ^+^ SN neurons projecting to the tail of the striatum; Cluster d: *Slc17a6* ^+^ VTA neurons projecting to the NAc shell, BLA, and PFC, alongside *Vgat* ^+^ VTA neurons projecting locally as well as to the NAc, PFC, and central amygdala; Cluster e: *Aldh1a1* ^+^/*Otx2* ^+^ neurons in the ventromedial VTA projecting to the NAc shell; and Cluster f: *Vip* ^+^ neurons located caudally in the PAG and DR that project to the central amygdala (Poulin et al., 2018; 2020). Among these molecularly defined subpopulations, Clusters d (*Slc17a6* ^+^ VTA) and f (*Vip* ^+^) appear uniquely relevant to stress susceptibility.

Beyond transcriptomic identity, target-derived molecules often use opposing signaling pathways to establish dopaminergic synapses in a circuit-specific manner. For example, Terauchi et al. (2023) found that two groups of antagonistic transforming growth factor- β (TGF- β ) family members—specifically bone morphogenetic protein 6/2 (BMP6/2) versus TGF- β2 —differentially coordinate dopaminergic synapse development. The BMP6/2–BMPR–Smad1 axis organizes nigrostriatal projections, whereas the TGF- β2 –TGF- β R–Smad2 axis regulates mesolimbic projections (Terauchi et al., 2023). Postnatally, knockout of these Smad proteins does not compromise cell survival, dendritic development, or axon targeting, suggesting that this signaling is required for presynaptic differentiation. Crucially, while *Smad1*-mutant mice exhibit motor deficits, *Smad2*-mutant mice display motivational deficits (Terauchi et al., 2023), suggesting that Smad2-mediated mesolimbic signaling may play a key role in mediating depression-like behaviors.

While recent advancements in single-cell sequencing technologies and expanded transcriptomic datasets have uncovered an even larger array of data-driven DA clusters (e.g., (*Slc6a3* (DAT) or *Slc18a2* with *Th* and *Ddc*) (Langlieb et al., 2023; Yao et al., 2023), it remains imperative to move beyond purely transcriptomic classification to rigorously validate the spatial, anatomical, and functional boundaries of these novel subtypes, particularly mapping these molecular cell-type signatures onto functional projection targets (e.g., VTA→NAc vs. VTA→PFC ) and distinct behavioral endophenotypes. Elucidating how transcriptomic cell-type identity dictates projection-specific physiological adaptations under stress represents a critical prerequisite for understanding phenotypic heterogeneity. How these discrete transcriptomic clusters modulate the diverse behaviors governed by mammalian DA neurons, and their specific causal contributions to stress susceptibility and the expression of distinct depression-like phenotypes, remain to be fully investigated. Furthermore, exposure to chronic stressors across critical developmental periods, such as infancy, childhood, and adolescence can irreversibly remodel macro-circuit architecture, onsetting psychiatric disorders including MDD. Investigating how chronic stress alters the trajectory of dopamine system maturation during these sensitive periods is therefore critical to understanding how early-life adversity causally shapes adult behavioral vulnerability.

### Is depression a dopamine-mediated disorder of blunted reward prediction error signaling?

Reward prediction errors (RPEs), the scalar discrepancy between expected versus predicted rewards, are cardinal for reinforcement learning. Blunted RPE signaling and impaired reward-driven learning disrupt the motivation to seek out rewarding experiences, providing a mechanistic basis for anhedonia in MDD. In depressed individuals, phasic reward-learning signals are significantly attenuated, manifesting as blunted neural activity within critical reward nodes (e.g., ventral striatum, hippocampus), that correlate directly with illness severity (Kumar et al., 2008). Behavioral and neuroimaging studies suggest that individuals with MDD exhibit severe deficits in processing both positive outcomes and error-correction signals. Unlike healthy controls, who dynamically adjust their reaction times and recruit the VS following positive feedback or the anterior cingulate cortex (ACC) following negative feedback, depressed individuals fail to flexibly adapt their behavior or properly engage these circuits (Steele et al., 2007). Notably, the magnitude of this behavioral rigidity correlates directly with self-reported anhedonia (Steele et al., 2007). Depressed individuals show blunted frontostriatal activity specifically during the anticipation of gains and losses. Importantly, while striatal encoding of positive RPEs is selectively blunted, the neural encoding of negative prediction errors often remains preserved or even heightened. Together, these findings suggest that disrupted positive RPE signaling and impaired reward-seeking motivation are core hallmarks of MDD, operating alongside an intact or biased capacity for negative associative learning.

The severity of reward-learning deficits in MDD is not only inextricably linked to anhedonic symptoms but also serves as a critical predictor of treatment failure. Patients who lack the computational and cognitive flexibility to dynamically update their behavior as a function of reward probability face a persisting diagnosis of MDD, with baseline reward-learning deficits increasing the odds of an ongoing MDD diagnosis following 8 weeks of standard treatment (Vrieze et al., 2013). Blunted reward responsiveness persists in adults with remitted depression, independent of any residual symptoms or acute stress exposure (Pechtel et al., 2013). This enduring impairment suggests that dysfunctional reward learning represents a stable, trait-like neurobiological vulnerability in MDD rather than a state-dependent artifact.

Molecular and functional neuroimaging studies directly link these reward-learning impairments to altered DA transmission and mesostriatal circuit dysfunction. Voxel-based PET analyses indicate a significant lower DAT availability in midbrain regions including the VTA and the SN regions, and in the right dorsal putamen in depressed patients (Dubol et al., 2020), pointing to a state of blunted baseline DA function. Notably, this dopaminergic dysfunction was exacerbated by recurring episodes of depression (Pizzagalli et al., 2019). Compared to healthy controls, individuals with MDD display blunted striatal RPE signals during both reward anticipation and delivery, alongside overall reduced functional connectivity within the VTA–striatal pathway during outcome feedback (Kumar et al., 2018; Geugies et al., 2022). Crucially, this attenuation of striatal RPE signaling scales with disease burden, becoming increasingly blunted in patients with a history of multiple episodes of depression (Kumar et al., 2018). Signal attenuation within mesostriatal circuits directly anchors the clinical loss of pleasure to a breakdown in fundamental reward-tracking mechanics: the extent of RPE signal reduction across the bilateral caudate, NAc, and midbrain directly correlates with increased self-reported anhedonia severity (Gradin et al., 2011). However, some heterogeneity exists, as individuals with mild-to-moderate depression can exhibit preserved ventral striatal RPE signals comparable to healthy controls (Rutledge et al., 2017). Recent computational accounts propose that mesocortical hyperconnectivity in MDD impairs the neural tracking of RPEs, driving distinct symptom dimensions (Beltrán et al., 2025). Specifically, blunted RPE signaling in the NAC predicts greater anhedonia, whereas blunted tracking within the BLA correlates with increased anxiety (Beltrán et al., 2025). Thus, aberrant mesocortical hyperconnectivity appears to disrupt core reinforcement learning computations in a circuit-specific manner, accounting for the clinical and symptomatic heterogeneity of depression.

Evidence from animals models strongly reinforces these clinical and computational paradigms. Within the NAc, DA terminals in ventral NAc medial shell (vNAcMed) are particularly excited by unexpected aversive outcomes and associated predicting cues, whereas DA terminals in other NAc subregions are inhibited by aversive stimuli (Jong et al., 2019). These stress-responsive DA→vNAcMed neurons receive aversive outcome signals from glutamatergic ( Vglut2 -expressing) neurons in the LH (Jong et al., 2019). Similarly, synapses on VTA DA neurons projecting to mPFC were modified only by the acute aversive stimulus (subcutaneous formalin injection) (Lammel et al., 2011). While classic electrophysiological studies noted that aversive stimuli such as tail pinches, air puffs, or foot shocks predominantly suppress VTA DA neuron activity over time during learning (Mirenowicz and Schultz, 1996), subsequent population recordings revealed notable functional heterogeneity: distinct subsets of VTA DA neurons are either inhibited or activated by noxious events (Brischoux et al., 2009; Butts and Phillips, 2013). This indicates that projection-specific DA ensembles differentially encode the salience, valence, and severity of both acute stressors and chronic environmental adversity.

Emerging conceptual frameworks suggest that classical RPEs may represent a specific manifestation of a broader, generalized arousal signal that governs biological predictive processing (Beck and Friedman, 2026; Burke et al., 2026; Gallello et al., 2026), which may become profoundly disrupted under chronic stress to precipitate depression-like behaviors. At the same time, recent work in computational psychiatry reinforces the premise that mesolimbic DA dysfunction driving depressive motivational and reward deficits reflects fundamental impairments in RPE signaling and value-guided decision-making (Botvinick et al., 2025). In particular, computational models highlight disruptions in distributional reinforcement learning (Lowet et al., 2020), which posits that the brain represents reward value not as a uniform point-estimate (expected mean), but as a full probability distribution encoded across transcriptomically and physiologically diverse populations of DA neurons (Botvinick et al., 2025). Unraveling how stress-induced disruptions in distributional reward processing induce anhedonia and related behavioral phenotypes requires rigorous pre- and post-stress evaluation across multiple cognitive and decision-making domains. Behavioral paradigms that probe nuanced cognitive features, such as the serial blocking effect (Mahmud et al., 2019), the restaurant row task (Cuttoli et al., 2024), and operant social reward-seeking procedures (Venniro and Shaham, 2020), will be critical for mapping these theoretical distributional encoding deficits onto specific clinical symptom dimensions.

### Animal models of stress-induced depression-like behaviors

Rodent stress paradigms, including Chronic Unpredictable Mild Stress (CUMS), Chronic Restraint Stress (CRS), and Chronic Social Defeat Stress (CSDS), are foundational paradigms for unraveling the cellular and molecular mechanisms underlying depression (Czéh et al., 2016; Wang et al., 2017; Mahmud et al., 2023; Mahmud and Karaman, 2026). A comprehensive evaluation of the translational utility, methodological strengths, and inherent limitations of these stress paradigms can be found in our prior reviews (Mahmud et al., 2023; Mahmud and Karaman, 2026). Collectively, these models induce a wide spectrum of behavioral and physiological deficits, such as anhedonia, cognitive impairment, hypothalamic-pituitary-adrenal (HPA) axis dysregulation, and prefrontal cortical dendritic atrophy (see Figure 1). While CUMS captures the essence of unpredictable daily human stressors (despite high inter-laboratory variability) and CRS offers strong reproducibility for studying structural neural remodeling (despite potential habituation), traditional CSDS excels at segregating susceptible from resilient phenotypes. Importantly, because standard CSDS relies on territorial male-to-male aggression, recent methodological adaptations in females, including chemogenetic aggression control (Takahashi et al., 2017), pheromonal provocation (e.g., male urine from CD-1 aggressors (Harris et al., 2018), and *fem*CSDS model (Patel et al., 2026) now allow researchers to rigorously investigate sex differences under comparable stress conditions.

In addition to adult stress models, paradigms targeting specific developmental windows such as early-life stress (ELS) and adolescent stress are vital for understanding how environmental adversity alters the developmental trajectory of midbrain dopamine circuits (Plotsky and Meaney, 1993; Spear, 2000; Meaney, 2001). ELS models such as maternal separation (MS) and limited bedding and nesting (LBN) induce persistent hyper-reactivity of the HPA axis, alters VTA pacemaking activity, and permanently impairs DAT and receptor expression in the NAc and PFC (Meaney, 2001; Peña et al., 2017). Stress exposure during adolescence via adolescent social defeat stress or adolescent subchronic variable stress disrupts the normative maturation of VTA-PFC dopamine projections and induces enduring affective and cognitive deficits in adulthood (Novick et al., 2011; Burke et al., 2013; Sheth et al., 2017).

The impact of stress on the mesolimbic dopamine system is highly nuanced, as distinct stressors can elicit opposing physiological responses from VTA dopamine neurons depending on prior exposure history, modality, timing, and severity (Rincón-Cortés and Grace, 2020). For example, while both CRS and CSDS induce depressive-like phenotypes by shifting the E/I balance toward hyper-excitation in medial preoptic area (MPOA) circuits, they target distinct cell populations (Tao et al., 2024). Under CRS, hyperactive MPOA glutamatergic neurons suppress midbrain dopamine and serotonin signaling via projections to the VTA and PAG/DR, respectively, to mediate specific depressive symptoms. In contrast, CSDS selectively blunts local inhibitory GABAergic activity within the MPOA and their activation lessens CSDS-induced depression-like behaviors (Tao et al., 2024). Furthermore, dopamine signaling serves divergent roles during the initial induction versus the subsequent phenotypic expression of depression-like behaviors, with the chronicity of the stressor directly scaling the magnitude and persistence of the negative affective state observed upon its cessation. Initial increase in DA activity could be an active process for adaptability to the changing environment and as a result of increased demands due to severity of the stressor(s), while the diminished DA activity after chronic stress may result in persistent anhedonia and motivational deficits.

Beyond its classical role in reinforcement learning and motivation, the dopaminergic system coordinates feeding behavior and energy balance by functioning as an integrated network across mesocorticolimbic and hypothalamic circuits that synthesize hedonic motivation, nutritional status, and peripheral homeostatic cues via cell-type-specific signaling (Onimus et al., 2026), processes and behaviors often disrupted by chronic stress. Consequently, evaluating post-stress alterations in feeding patterns and energy expenditure and balance can provide critical insights that extend beyond traditional behavioral assessments.

It should be noted that preclinical (rodent) paradigms such as CSDS or CUMS model specific stress-induced behavioral alterations (e.g., social avoidance, impaired cognitive flexibility) capture isolated dimensional endophenotypes rather than the full, complex diagnostic spectrum of human depression. Similarly, while targeted neural manipulations such as optogenetics and chemogenetics provide powerful tests of pathway causality, gain-of-function, and circuit sufficiency, artificial high-frequency light stimulation often induces highly synchronized neuronal ensemble firing that does not reflect endogenous, physiological dynamics. Likewise, biophysical properties such as alterations in $I_{h}$ or DAT expression vary dynamically across acute stress adaptation versus prolonged defeat states (Friedman et al., 2014; Grace, 2016). For instance, decreased DAT expression does not simply reflect reduced extracellular dopamine release; rather, it may also signify altered membrane clearance kinetics, transporter redistribution, or local presynaptic terminal remodeling.

### Chronic stress, dopamine, and senescence

Chronic stress significantly accelerates biological ageing (Poganik et al., 2023). By integrating transcriptomes across four mammalian species (including rodents and humans), a comprehensive cross-species study identified universally conserved biomarkers of chronological age, most notably *CDKN1A* and *LGALS3*, that reliably predict time to death, age-related multimorbidity, and the efficacy of rejuvenating interventions (Tyshkovskiy et al., 2026). Interestingly, inhibiting Galectin-3 (Gal-3, encoded by *LGALS3*) may prevent the loss of dopaminergic neurons by attenuating the accumulation of α-synuclein and the subsequent upregulation of proinflammatory cytokines via microglial activation (Boza-Serrano et al., 2014). As a potent neuroinflammatory amplifier, Gal-3 orchestrates a proinflammatory microenvironment that compromises dopaminergic synaptic integrity and induces the maladaptive plasticity underlying depression-like phenotypes (Mijailović et al., 2022; García-Revilla et al., 2023). The vulnerability of dopaminergic circuits to chronic stress is further mediated by the upregulation of *CDKN1A* (p21), which induces premature cellular senescence. Due to the high metabolic demands of dopamine synthesis and packaging, DA neurons are intrinsically vulnerable to stress-induced oxidative damage. Prolonged physiological stress activates the p53/p21 DNA damage response pathway and upregulates *CDKN1A*, driving post-mitotic dopaminergic neurons into a senescent-like phenotype (Martínez-Cué and Rueda, 2020). Crucially, this *CDKN1A*-mediated senescence fundamentally impairs synaptic plasticity and triggers the release of senescence-associated secretory phenotype (SASP) factors (Kritsilis et al., 2018). The secretion of these SASP factors into the extracellular space may disrupt local DA transmission and catalyze the aforementioned microglial activation, creating a feed-forward loop of neuroinflammation and dopaminergic dysfunction that may underlie depression-like behaviors.

Emerging evidence indicates that stress-induced sleep disturbances and circadian misalignment can further accelerate cellular senescence within dopaminergic pathways. Stress activates a subset of VTA GABA-somatostatin ( $VTA^{Vgat-Sst}$ ) neurons that induce non–rapid eye movement (NREM) and REM sleep via the lateral hypothalamus while concurrently suppressing corticotropin-releasing factor (CRF) release in the paraventricular hypothalamus (Yu et al., 2022). Ablating this cell population eliminates stress-induced sleep, leaving anxiety and corticosterone concentrations elevated (Yu et al., 2022), underscoring their essential role in sleep-mediated physiological recovery. Remarkably, these VTA GABA-somatostatin neurons inhibit neighboring DA neurons (Nagaeva et al., 2020). Chronic stress-induced sleep disturbances may increase cellular senescence by compounding DNA damage, shortening telomeres, and accelerating epigenetic aging (Carroll and Prather, 2021). These sleep deficits are tied to localized disruptions in circadian mechanisms that destabilize mesolimbic dopamine dynamics and mood stability. For instance, mice with a mutation in the circadian *Clock* gene (*ClockΔ19*) exhibit rapid mood-cycling, aberrant daytime spikes in VTA dopaminergic activity, tyrosine hydroxylase (TH) expression, and overall dopamine synthesis, a phenotype recapitulated by sustained optogenetic VTA activation and reversed by time-dependent TH suppression (Sidor et al., 2015). This reciprocal circadian-dopamine crosstalk is directly implicated in stress pathology: CSDS induces hypersomnia and abnormal NREM electroencephalogram spindles via hyperactivation of the VTA-mPFC DA pathway (Li et al., 2026). Chemogenetic silencing of this VTA-mPFC DA pathway during CSDS or the post-stress pharmacological inhibition of hyperpolarization-activated *I*_h_ currents in VTA neurons rescues these sleep disturbances (Li et al., 2026). Taken together, these findings reveal a destructive feedback loop: stress-induced dopaminergic hyperactivity destabilizes sleep and circadian rhythms, which may in turn fuel the accumulation of DNA damage and accelerate cellular senescence within midbrain reward circuitry.

Exposure to chronic stress triggers profound, circuit-specific remodeling of the dopaminergic system, characterized by distinct functional, morphological, and transcriptional alterations across sex and pathway boundaries. Below, we review seminal and recent studies detailing VTA projections to key reward nodes, highlighting how maladaptation within these specific circuits precipitates and maintains depressive-like behaviors.

### Alterations in VTA DA neurons

In a seminal paper, Berton and colleagues (2006) showed that brain-derived neurotrophic factor (BDNF) signaling in VTA DA neurons is functionally required for the emergence of experience-dependent social avoidance following CSDS (Berton et al., 2006). Viral-mediated, pathway-specific knockdown of BDNF in mesolimbic dopamine projections obliterates the widespread transcriptomic remodeling within the NAc that is normally induced by repeated aggression. Crucially, chronic antidepressant treatment mimics this transcriptomic rescue (Berton et al., 2006), reinforcing the predictive validity and translational utility of the CSDS model. Subsequently, Krishnan et al. (2007) reported an increased firing rate specifically in VTA DA neurons in susceptible mice compared to resilient mice after CSDS. Notably, resilient mice exhibit more genes regulated in the VTA compared to susceptible mice, characterized by the upregulation of several voltage-gated potassium ( $K^{+}$ ) channel subunits (Krishnan et al., 2007). Targeted knockdown of $K^{+}$ channel regulatory subunits (e.g., *Kcnab2*) in naïve mice augmented the firing rate of VTA DA neurons and induced susceptibility, even after a sub-threshold CSDS (three defeats in 1 day) (Krishnan et al., 2007). These findings suggest that stress resilience is not merely a passive absence of vulnerability, but an active neurobiological process mediated by intrinsic homeostatic adaptations, specifically the recruitment of $K^{+}$ channels to stabilize VTA DA excitability. Building on these mechanisms, CSDS elevates VTA DA neuron spontaneous firing, burst activity, and hyperpolarization-activated cation currents ( $I_{h}$ ) in susceptible mice, alterations that track closely with social avoidance behaviors (Cao et al., 2010). These behavioral and neurophysiological deficits are fully reversed by chronic (but not acute) fluoxetine administration, while local VTA pharmacological blockade of $I_{h}$ channels replicates this antidepressant action (Cao et al., 2010), demonstrating that mesolimbic dopamine firing dynamics mediate both stress vulnerability and therapeutic recovery. However, recent studies suggest that stress-induced loss of postsynaptic density protein 95 (PSD-95) may account for disruptions in BDNF signaling and impaired structural plasticity (Shi et al., 2024; Bevilacqua et al., 2026).

Beyond firing dynamics, VTA DA activity-regulated myelin plasticity plays a critical role in structural circuit modification and reward learning (Yalçın et al., 2024). Chronic stress induces myelin loss in corpus callosum via a mechanism that couples Wnt/β‐catenin pathway activity with dopamine $D_{2}$ receptor ( $D_{2}R$ ) signaling (Choi et al., 2017). Single-nucleus RNA sequencing further confirms that stress resilience is orchestrated by active, widespread transcriptional remodeling within the VTA, whereas the LHb remains molecularly stable (Minerva et al., 2026). Across VTA cell types, particularly dopaminergic and glutamatergic clusters, resilience is characterized by the upregulation of genes regulating intercellular signaling and neural communication via maintenance of receptor-ligand interaction strength (Minerva et al., 2026). Importantly, transcriptional profiles in resilient animals diverge more significantly from non-stressed controls and susceptible animals (Minerva et al., 2026), suggesting that resilience represents an active, hyper-adaptive molecular adaptation. Finally, the temporal dynamics of stress relief provide a critical lever for behavioral fate. The magnitude of relief experienced immediately following physical stress termination strongly correlates with individual resilience to depression-like behaviors, a phenomenon may contribute to the persistent, minute-long activation of mesolimbic dopamine neurons (Dong et al., 2023). Manipulating downstream VTA targets (DA projections to NAc lateral shell and dorsomedial shell) during this transient relief window bidirectionally regulates behavioral outcomes: disrupting DA signaling during relief induces vulnerability, whereas augmenting its positive valence with natural rewards immediately post-stress enhances resilience (Dong et al., 2023). This unveils a proactive neurobiological mechanism and therapeutic window to counteract stress-induced affective pathology.

An apparent paradox in stress neurobiology lies in the divergent electrophysiological profiles reported across rodent stress paradigms: CSD reliably induces VTA DA hyperactivity in susceptible mice (characterized by increased burst firing and elevated $I_{h}$ currents; Krishnan et al., 2007; Chaudhury et al., 2013), whereas CUMS and CMS induce VTA DA hypoactivity and blunted extracellular dopamine tone (Grace, 2016; Belujon and Grace, 2017). This discrepancy can be reconciled by considering three critical distinctions: projection-specific divergence, spontaneous firing rate vs. population activity, and ethological nature of the stressor. First, CSDS hyperactivity is restricted primarily to mesoaccumbens ( VTA→NAc ) projecting neurons, where it induces social avoidance. In contrast, mesocortical ( VTA→mPFC ) projecting DA neurons in the same defeated animals exhibit mostly hypoactivity and blunted firing rates, suggesting that CSDS does not simply produce indiscriminate, global VTA hyperactivity (Lammel et al., 2012; Chaudhury et al., 2013). Second, *in vivo* electrophysiological studies show that while CSDS elevates the firing rate and bursting pattern of individually recorded active DA neurons, CUMS mainly impairs VTA DA population activity (Grace, 2016). CUMS-induced stress also heightens baseline GABAergic drive from the VP and RMTg, driving large subsets of VTA DA neurons into a hyperpolarized state, even if the remaining active cells show altered intrinsic conductance. Finally, CSDS models an acute-on-chronic, ethologically relevant social threat that requires continuous vigilance and defensive appraisal. This elevated threat salience drives hyperactive phasic bursting. Conversely, CUMS relies on prolonged, non-social, inescapable physical and environmental stressors (e.g., circadian inversion, cage tilting, wet bedding). This persistent, uncontrollable exposure triggers allostatic exhaustion and learned helplessness, eroding baseline motivational drive and blunting phasic RPE output to induce depression-like phenotypes.

### Morphological and sex-specific alterations in midbrain dopamine circuits

Beyond functional electrophysiological alterations, chronic stress induces significant morphological and cellular remodeling within midbrain DA pathways. Mice susceptible to CSDS exhibit a significant, mammalian target of rapamycin complex 2 (mTORC2) signaling-dependent reduction in the soma size of VTA dopamine neurons (Kaska et al., 2017), whereas CRS in rats triggers a distinct histopathological loss of DA neurons within both the VTA and the hypothalamus (Sugama and Kakinuma, 2016). Stress-induced structural remodeling is highly selective across sub-populations: following CSDS, spine density within the NAc and the VTA is selectively elevated in susceptible cohorts compared to control and resilient mice (Qu et al., 2018). At the receptor level, glucocorticoid receptor (GR) signaling in dopaminoceptive target cells, rather than DA-releasing neurons, specifically induce social avoidance without altering anxiety phenotypes or fear memory consolidation (Barik et al., 2013). Crucially, acute optogenetic or pharmacological inhibition of these DA-releasing projection neurons is sufficient to restore normal social interaction behaviors in socially defeated wild-type mice (Barik et al., 2013). Taken together, these findings suggest that the specific modality, duration, and severity of chronic stress dictate highly localized, differential, and potentially permanent structural and cellular adaptations across dopaminergic neurons.

Chronic stress also orchestrates complex multi-transmitter adaptations across distributed forebrain networks. A defining consequence of chronic stress is the disruption of established social ranking, with VTA DA signaling serving as a critical mediator of this stress-induced social reorganization. While higher baseline sociability predicts higher social rank in mice and characterized by enhanced working memory, elevated anxiety, and suppressed VTA activity that confers resilience to stress, pharmacogenetic inhibition of VTA DA neurons or genetic deletion of glucocorticoid receptors in downstream dopamine-responsive targets causally facilitates entry into higher social ranks (Battivelli et al., 2024), providing a plausible framework for midbrain dopaminergic tone as a crucial determinant of social dominance and competitiveness under stress. A four-week CUMS induced multiple behavioral deficits and associated dopaminergic hyperfunction and elevated ERK/Akt/CREB signaling cascades within the NAc, alongside concurrent serotonergic hypofunction and diminished TH expression across the hippocampus and PFC, effects that were mitigated by daily treatment with either fluoxetine or aripiprazole during the stress (Lu et al., 2019). At the intrinsic microcircuit level, selective downregulation of transient receptor potential canonical 6 (TRPC6), a calcium-permeable non-selective cation channel, in VTA DA neurons depresses the activity of NAc-projecting dopaminergic neurons and contributes to CUMS-induced depressive phenotypes (Wang J. et al., 2024). Similarly, exposure to the chronic unpredictable mild stress/restraint (CUMR) impairs weight gain, induces social withdrawal, and paradoxically elevates dominant behavior (Mograbi et al., 2025). These effects are associated with diminished serotonin (5-HT) concentrations within the hippocampus while simultaneously augmenting DA levels within both the amygdala and hypothalamus, alongside accelerating amygdalar 5-HT turnover (Mograbi et al., 2025). Together, these findings indicate that complementary and compensatory neurotransmitter systems interact dynamically within distinct subcortical circuits to orchestrate stress-induced behavioral profiles.

Changes in social exploration motivation after chronic stress may be orchestrated by two opposing DA circuits. Tonic firing within a DA-D2R indirect pathway involving the mPFC is actively reinforced by positive social contact, while avoidant behavior and risky vigilance are encoded by phasic firing within a competing DA-D1R direct pathway (Zheng et al., 2026). The shifting equilibrium between these two distinct DA networks dynamically dictates the final behavioral decision to either explore or avoid a given environment. Although, considerable sex differences exist in baseline and stress-induced DA dynamics and function in the VTA (Gillies et al., 2014; Bale and Epperson, 2015; Becker and Chartoff, 2019; Rainville et al., 2022), the specific mechanisms governing stress-induced DA dysfunction in females remain an active area of investigation. Following chronic mild stress, activity of VTA DA neurons was reduced more in females (≈ 75%) than males (≈ 50%) (Rincón-Cortés and Grace, 2017). Dopamine signaling in non-neuronal cell populations also play a critical, sex-divergent role: microglia and complement-mediated phagocytic activity shape adolescent NAc development by eliminating dopamine D1 receptors in male, but not female, rats (Kopec et al., 2018). Following CUS, mPFC levels of β2 adrenoceptors and D1 receptors were reduced in male rats, but not in females (Jankovic et al., 2022). Chronic social defeat in female mice induces VTA DA hyperactivity exclusively in stress-susceptible subjects, a pathophysiological state reversed by a single dose of ketamine and underwired by an elevated cholinergic tone from the LDTg (Ortiz et al., 2022). Resilient females evade this susceptibility through a protective insensitivity to nicotinic inputs, and *in vivo* activation of VTA α7 nicotinic acetylcholine receptors enhances dopaminergic firing and forces a phenotypic switch from resilient to susceptible (Ortiz et al., 2022), suggesting these receptors as key molecular substrates gating female stress vulnerability. Following subchronic variable stress (SCVS), female, but not male, mice exhibit a reduced *ex vivo* firing rate in VTA DA neurons, but both sexes show an increase in the firing rate of VTA GABAergic neurons (Bouarab et al., 2025). Interestingly, male mice exhibit a compensatory increase in excitatory synaptic tone onto VTA DA neurons that is absent in females (Bouarab et al., 2025), suggesting a male-specific circuit adaptation that buffers against SCVS-induced behavioral deficits.

In female rodents, circulating estrogen sensitizes the stress response system by amplifying both noradrenergic and dopaminergic signaling (Bangasser and Valentino, 2012). This hormone-induced elevation in catecholaminergic activity lowers the neurobiological threshold for stress reactivity, rendering these neural circuits inherently more vulnerable to subsequent environmental challenges. For example, inhibiting VTA estrogen receptors during diestrus mimics the heightened stress vulnerability typical of estrus, whereas increasing local potassium channel activity prior to stress rescues social interaction deficits in female mice in estrous phase (Shanley et al., 2023). This vulnerability may be due to stress-induced changes in RPE and reinforcement learning dynamics. Elevated 17β-estradiol downregulates NAc core DAT expression, prolonging extracellular dopamine availability and strengthening the RPE signals that govern effort-reward trade-offs (such as trial initiation speed) (Golden et al., 2025). Midbrain estrogen receptor knockdown abolishes this reward sensitivity, confirming that endogenous estrogen dynamics causally dictate reward-guided decision-making through circuit-level modulation of mesolimbic dopamine reuptake (Golden et al., 2025). Recent studies also show that the role of estrogen in gating stress reactivity extends far beyond peripheral gonadal hormone fluctuations as estrogen is synthesized locally within forebrain and limbic networks in both males and females where central estrogen levels directly shape structural synaptic plasticity and chromatin accessibility (Hokenson et al., 2021; 2026).

### The VTA-NAc circuit

Chronic stress significantly disrupts the NAc, a brain region associated with reinforcement learning and motivation, often by attenuating dopaminergic activity and impairing synaptic plasticity (Spring et al., 2021; Zhang et al., 2024; Lucantonio et al., 2025). Following CSDS, susceptible mice exhibit hyperactivity in the VTA DA neurons, accompanied by an upregulated *I*_h_, whereas resilient mice maintain normal neuronal firing rates despite exhibiting an even larger *I*_h_, a paradox explained by a compensatory upregulation of potassium (K^+^) channel currents (Friedman et al., 2014). Strikingly, exaggerating this *I*_h_ or optogenetically increasing the hyperactivity of VTA DA neurons in susceptible mice ameliorates depression-like behaviors (Friedman et al., 2014). This counterintuitive antidepressant effect is triggered by a projection-specific homeostatic plasticity (in VTA-NAc projection, but not in VTA-mPFC pathway) that mirrors the intrinsic adaptations of natural resilience (Friedman et al., 2014). At the terminal level, following CSDS, the density of TH^+^ dopaminergic axonal projections and expression of activity-dependent marker phosphorylated ERK (pERK) are decreased in the mPFC of susceptible male and female mice, whereas resilient animals are spared. Conversely, pERK expression is elevated within the NAc across both sexes, without concomitant changes in c-Fos expression (Quessy et al., 2021).

By integrating machine learning-based pose tracking (via DeepLabCut) with projection-specific fiber photometry, fast-scan cyclic voltammetry, and closed-loop optogenetic manipulations during live defeat encounters, Willmore and colleagues (2022) revealed that resilient and susceptible mice deploy highly divergent behavioral strategies during social stress encounters, encoded by distinct DA transients within NAc terminals (but not the tail striatum) (Willmore et al., 2022). Resilient mice exhibit elevated DA activity in close proximity to the aggressor and at the onset of fighting back, whereas susceptible animals display heightened DA signaling at the offset of attacks and the onset of fleeing. Randomly timed optogenetic stimulation of NAc-projecting DA neurons during social defeat and fighting-back-timed activation induce resilience and reorganize active defensive strategies toward resilient phenotypes (Willmore et al., 2022). Collectively, these data provide a plausible framework that time- and projection-specific dopamine transients dynamically and causally encodes individual differences in stress susceptibility versus resilience.

While chronic pain and depression frequently co-occur, distinct cell-type-specific pathways within the NAc mediate these conditions in nerve-injured models. The glutamatergic inputs from the anteromedial thalamic nucleus to NAc medial shell D2 neurons that innervate orexin-expressing neurons in the lateral hypothalamic area regulate pain sensitivity, while the lateral septum GABAergic inputs to NAc medial shell D2 neurons that disinhibit the ventral pallidum glutamatergic neurons controls depression-like behaviors (Liu et al., 2025). Cell-type-specific plasticity also determines susceptibility following social defeat. CSDS-induced depression-like behaviors are associated with decreased excitatory input frequency onto $D_{1}$ -MSNs (medium spiny neurons) and increased frequency onto $D_{2}$ -MSNs (Francis et al., 2015). While optogenetic stimulation of $D_{1}$ -MSN promotes resilience and inhibition induces depressive outcomes, modulating $D_{2}$ -MSNs does not alter behavioral responses to acute CSDS but instead primes stress-naive mice for social avoidance following subthreshold defeat (Francis et al., 2015). Chronic unpredictable stress triggers depressive-like phenotypes by reducing dopamine levels in the NAc through D2R-mediated presynaptic negative feedback. Pharmacological blockade of NAc D2Rs or direct dopamine infusion reverses these behavioral and neuronal deficits, whereas local D2R activation mimics stress-induced despair in stress-naïve animals (Qiao et al., 2020). Structural remodeling of these inputs extends to guidance receptor signaling. Following CSDS, Netrin-1/DCC (deleted in colorectal cancer) receptor signaling influences stress susceptibility by remodeling the apical dendritic architecture in PFC pyramidal neurons, particularly those projecting to the NAc shell (Mahmud et al., 2025), a key neural substrate for stress-induced dopamine release. Localized autophagy, the primary cellular recycling pathway, may serve as a parallel mechanism for this structural remodeling (Bouhidel et al., 2015). Indeed, a recent study showed that localized autophagic activity within the growth cones of developing midbrain dopamine neurons is critical for proper axonal branching and navigation; the deletion of essential autophagy genes produces structural abnormalities and abolishes the neuron’s ability to respond to environmental guidance cues (Profes et al., 2026). Importantly, the role of DCC receptors in female VTA DA neurons following chronic stress requires further investigation. Because DCC receptors are heavily enriched in VTA DA neurons and mediate sex differences across other psychiatric conditions (Hernandez et al., 2025), clarifying their role may yield critical mechanistic insights into sex-dependent stress susceptibility.

### The VTA-PFC circuit

The PFC serves as the primary neural substrate for high-order executive function, cognitive control, decision-making, long-term planning, and top-down affective regulation. Clinical and preclinical studies indicate that the therapeutic efficacy of classical and rapid-acting antidepressants relies heavily on their capacity to normalize PFC DA signaling. For example, chronic administration of selective serotonin reuptake inhibitors (SSRIs) such as fluoxetine stimulates dopamine release within the PFC and NAc while suppressing baseline VTA dopaminergic firing via elevated central serotonin ( 5‐HT ) levels, thereby resolving affective deficits tied to dopaminergic hypofunction (Prisco and Esposito, 1995; Ichikawa and Meltzer, 1999; Dunlop and Nemeroff, 2007). Recent studies also implicate PFC D2R + neurons in modulating depressive-like behaviors and mediating the effects of mechanistically distinct antidepressants, such as ketamine and fluoxetine, often in a precise, pathway-specific manner (e.g., mPFC-VTA and mPFC-NAc projections) (Wilke et al., 2022; Chen H. et al., 2024). Furthermore, ketamine-induced mPFC spinogenesis requires D1 receptor activation and postsynaptic protein kinase A (PKA) activity (Wu et al., 2021a). Notably, chemogenetic blocking of local PFC dopamine release or VTA dopaminergic transmission is sufficient to prevent ketamine’s action in the mPFC (Wu et al., 2021a; 2021b).

PFC top-down executive and affective control relies on the fine-tuning of local E/I balance, orchestrated by three classes of GABAergic interneurons: parvalbumin-expressing ( ${\text{PV}}^{+}$ ) fast-spiking interneurons, somatostatin-expressing ( ${\text{SST}}^{+}$ ) dendritic-targeting interneurons, and vasoactive intestinal peptide-expressing ( ${\text{VIP}}^{+}$ ) disinhibitory interneurons (Tremblay et al., 2016; Ferguson and Gao, 2018). Dopamine released from mesocortical terminals binds to distinct receptor subtypes across these interneuron subclasses to regulate microcircuit synchrony. Dopamine $D_{1}\text{Rs}$ are highly expressed on ${\text{PV}}^{+}$ interneurons, and their activation enhances intrinsic excitability and strengthens perisomatic inhibitory currents onto pyramidal projection neurons (Gorelova et al., 2002; Domenico and Mapelli, 2023). This feed-forward perisomatic inhibition improves the signal-to-noise ratio by suppressing non-specific background firing, and paces local gamma oscillations essential for working memory, cognitive flexibility, and active coping (Sohal et al., 2009). Conversely, ${\text{SST}}^{+}$ interneurons target distal apical dendrites of pyramidal neurons, providing feedback inhibition that limits recurrent excitation and gates inputs from the thalamus and the BLA (Riedemann, 2019). Dopamine acts via both $D_{1}$ and $D_{2}$ receptors on ${\text{SST}}^{+}$ neurons to filter out dendritic calcium spikes, setting input-specific thresholds (Izquierdo-Claros et al., 2000; Eban-Rothschild et al., 2016; Anastasiades et al., 2019). Finally, ${\text{VIP}}^{+}$ interneurons preferentially innervate ${\text{SST}}^{+}$ and ${\text{PV}}^{+}$ cells, establishing a disinhibitory circuit. Dopaminergic activation of ${\text{VIP}}^{+}$ interneurons releases pyramidal projection neurons from dendritic inhibition, allowing processing of salient reward and punishment signals (Pi et al., 2013; Anastasiades et al., 2019). Under chronic stress, blunted $D_{1}R$ signaling, coupled with stress-induced dendritic retraction in ${\text{PV}}^{+}$ and pyramidal neurons, may degrade ${\text{PV}}^{+}$ -mediated perisomatic inhibition, thus destabilizing cortical E/I balance and impairing the PFC’s capacity to exert top-down inhibitory control over subcortical limbic regions.

The functional impact of VTA dopamine neurons on stress susceptibility is dictated by the nature of the stressor, precise neuronal firing patterns (phasic versus tonic), and target-specific projection pathways. Phasic optogenetic activation of the VTA-NAc DA pathway induces susceptibility to CSDS, whereas its inhibition promotes resilience (Chaudhury et al., 2013). Conversely, activation of the VTA-mPFC projection does not induce susceptibility, yet its inhibition orchestrates a susceptible phenotype (Chaudhury et al., 2013), underscoring a profound circuit-specificity in the response to social stress. Furthermore, altering the activity of defined midbrain dopamine populations can bidirectionally control multiple depression-like behaviors induced by CMS while simultaneously shifting how these behaviors are encoded within the NAc (Tye et al., 2013). Dissecting these output-specific roles, Liu and colleagues (2018) showed that unpredictable CMS selectively depresses the firing activity of VTA-mPFC projecting neurons without altering VTA-NAc neurons. Crucially, their work revealed a distinct molecular and circuit segregation: the amelioration of depressive-like behaviors depended on blocking BDNF signaling in the mPFC, whereas comorbid nociceptive responses were regulated by BDNF signaling within the NAc shell (Liu et al., 2018). Prefrontal dopaminergic transmission, particularly in the prelimbic cortex (PrL) may be critical for the maintenance of normal dendritic morphology, which is often disrupted by chronic stress. Blockade of D1R in the PrL prevented restraint stress-induced apical dendritic retraction, compared to stress-naïve rats. Interestingly, D1R blockade induced dendritic retraction in stress-naïve rats, without altering plasma corticosterone levels (Lin et al., 2015), suggesting that alteration due to DA transmission, rather than HPA axis response may be involved in this process. Following CSDS, D1R expression is downregulated in the mPFC of susceptible mice, although knockdown of D1R in the mPFC in whole neuronal populations or excitatory neurons increased social avoidance behavior (Shinohara et al., 2018). Collectively, these findings highlight the complex, projection-specific adaptations of VTA dopamine neurons and suggest that distinct dopaminergic subpopulations differentially modulate behavioral outcomes based on stress history, modality, and chronicity.

### The VTA-BLA circuit

Historically studied for its role in fear and anxiety, the BLA, a limbic brain region involved in emotional processing receives direct dopaminergic innervation from the VTA. Dissecting this pathway following CSDS using *ex vivo* electrophysiology, *in vivo* fiber photometry, and projection-specific optogenetics, Morel and colleagues (2022) showed that the VTA-BLA dopamine circuit selectively controls the expression of anxiety-like, but not depressive-like behaviors and may contribute to a causal link between the activity of these DA neurons and anxiety-like behaviors (Morel et al., 2022). Subsequent study showed that rather than merely tracking reward magnitude or associative strength, BLA dopamine signaling responds robustly to highly salient environmental cues regardless of their positive or negative emotional valence (Brickner et al., 2026).

### The VTA-DRN circuit

Beyond classic serotonergic outputs, the dorsal raphe nucleus (DRN) engages in complex multi-transmitter interactions with midbrain reward circuits. Activation of a glutamatergic pathway originating from VGluT3-expressing (vesicular glutamate transporter 3) neurons in the dorsal raphe modulates reward and reinforcement behaviors by directly exciting mesolimbic DA neurons in the VTA via AMPA (α-amino-3-hydroxy-5-methyl-4-isoxazolepropionic acid) receptors, triggering downstream dopamine release in the NAc (Qi et al., 2014). This architecture highlights functional interplay among midbrain dopaminergic, GABAergic, and glutamatergic networks. In CUS model, depression-related behaviors are modulated by a selective suppression of excitatory synaptic transmission onto DRN dopamine neurons. This stress-induced deficit is regulated by a specific DRN projection to the dorsal BNST, where dopamine acts via D2 receptors to modulate NMDAR-mediated excitatory currents in local CaMKII + target neurons (Wang W. et al., 2024). In addition to chronic stress models, acute social isolation-induced synaptic changes are observed in the DA neurons in the DRN (Matthews et al., 2016). Utilizing *in vivo* calcium imaging, researchers showed that DRN DA neurons show increased activity upon social contact following isolation. Optogenetic activation of these neurons increased social preference but also induced place avoidance, whereas inhibition suppresses a “loneliness-like” state (Matthews et al., 2016). Crucially, the behavioral impact of optogenetic manipulation of DRN DA neurons is pre-determined by the animal’s baseline social rank (Matthews et al., 2016). These social isolation-induced microcircuit adaptations and motivational shifts may be modulated by regional downregulation of *Bdnf* expression within midbrain-raphe networks (Lei et al., 2026).

### The VTA-vHIPP circuit

A fundamental paradox in the neurobiology of stress resides in how distinct stress modalities alter midbrain DA physiology. While multiple animal models of depression, including CMS, chronic cold stress, and learned helplessness, induce a significant downregulation in the population of spontaneously active DA neurons within the medial VTA, susceptibility to CSDS is paradoxically induced by elevated baseline and phasic burst firing rates among these same dopaminergic projections, suggesting that distinct stress pathologies may express unique firing dynamics (firing rate versus the total pool of active, pacemaking neurons) or represent a mechanistic boundary separating the latent susceptibility to a depressive phenotype from its active symptomatic expression (Grace and Uliana, 2026). The ventral hippocampus (vHIPP) may act as a central limbic hub orchestrating this homeostatic balance. Acute stress initially triggers the vHIPP to upregulate VTA dopamine neuron firing, an effect normally counterbalanced and normalized over time via reciprocal inhibitory control exerted by the amygdala (Belujon and Grace, 2017). However, under conditions of persistent stress-induced amotivation, the long-term potentiation (LTP) characterizing the hippocampal-accumbens pathway is pathologically transitioned into a state of long-term depression (LTD) (Belujon and Grace, 2014). Crucially, the rapid antidepressant efficacy of ketamine and its capacity to reverse anhedonia rely directly on rescuing this disrupted hippocampal-accumbens LTP and restoring the spontaneously active VTA dopaminergic population (Lucantonio et al., 2025).

### The VTA-LHb circuit

The lateral habenula (LHb) serves as an “anti-reward” center that modulates midbrain monoaminergic output. In learned helplessness models of depression, presynaptic potentiation at excitatory synapses onto VTA-projecting LHb neurons mediates an animal’s helplessness behaviour, and depletion of transmitter release by repeated electrical stimulation of LHb afferents rescues learned helplessness (Li et al., 2011). Using single-unit extracellular recordings, researchers evaluated the impact of infralimbic (IL) PFC versus LHb modulation on VTA dopamine neurons following 5–7 weeks of CMS. Stimulating the IL PFC specifically targeted the medial VTA neurons that are vulnerable to CMS, whereas LHb stimulation targeted the stress-resilient lateral VTA. Furthermore, only selective inactivation of the IL PFC, but not the LHb was sufficient to rescue dopamine population activity in stressed animals (Moreines et al., 2017), highlighting a unique top-down prefrontal mechanism for gating midbrain dopamine tone in chronic stress. Beyond general habenular hyperactivity, chronic restraint stress elevates the activity of $D_{1}R$ -expressing glutamatergic neurons in the LHb, whose optogenetic activation, either somatically or at their terminals within the VTA, is sufficient to precipitate behavioral despair and anhedonia (Chen X. et al., 2024). Conversely, optogenetic inhibition of this $LHb^{D_{1}R}\to \text{VTA}$ circuit rescues these stress-induced depressive phenotypes (Chen X. et al., 2024), establishing this specific habenulo-mesolimbic sub-circuit as a potential therapeutic target for stress-induced affective phenotypes.

Notably, LHb projection neurons are exclusively glutamatergic; thus, LHb hyperactivation does not directly inhibit VTA DA neurons. Instead, this inhibition is disynaptically mediated via the RMTg (Jhou et al., 2009; Hong et al., 2011). Descending glutamatergic projections from the LHb densely innervate the RMTg, forming strong excitatory synapses onto RMTg GABAergic neurons. Once activated, these RMTg neurons send robust, monosynaptic ${\text{GABA}}_{A}$ - and ${\text{GABA}}_{B}$ -receptor-mediated inhibitory inputs onto mesoaccumbens and mesocortical VTA DA neurons (Proulx et al., 2014). Additionally, a minority of LHb collaterals terminate within the VTA on GABAergic interneurons, recruiting feed-forward inhibition (Oriol et al., 2025). Under conditions of chronic stress, continuous hyperactivity of the LHb-RMTg pathway delivers tonic GABAergic braking onto VTA DA neurons, blunting phasic RPE transients and disrupting active coping behaviors.

### The VTA-LC circuit

Locus coeruleus (LC) noradrenergic inputs to the VTA play a key role in dictating stress resilience versus susceptibility. LC-derived norepinephrine transmission to the VTA is both necessary and sufficient to promote behavioral resilience by suppressing the hyperexcitability of NAc-projecting DA neurons (Isingrini et al., 2016). Following CSDS, LC inputs to the VTA determine stress outcomes, exhibiting enhanced firing activity in resilient, but not susceptible mice. Optogenetically mimicking this activity in susceptible mice reverses depression-like behaviors by normalizing cellular hyperactivity and restoring homeostatic plasticity in VTA-NAc dopamine neurons (Zhang et al., 2019). This pro-resilient effect is accompanied by α1-and β3-adrenergic receptors enriched on these mesoaccumbens neurons; antagonism of these receptors blocks the therapeutic benefits of LC-VTA stimulation (Zhang et al., 2019).

Beyond noradrenergic regulation, mesopontine cholinergic and GABAergic inputs calibrate VTA dopaminergic tone during chronic stress. CSDS induces VTA DA neuron dysregulation and depressive-like behaviors by triggering hyperactivity in cholinergic LDTg neurons projecting to the VTA (Fernandez et al., 2018). This pathological circuit remodeling is mediated by local neuroendocrine signaling through corticotropin-releasing factor receptor 1 ( CRF‐R1 ), the blockade of which or the direct chemogenetic inhibition of the LDTg→VTA pathway rescues the stress-induced phenotype (Fernandez et al., 2018). Conversely, susceptible animals exhibit hyperactivity of VTA DA neurons alongside an upregulation of local GABAA receptors (Ma et al., 2026). Dampening this dopaminergic hyperexcitability, either via direct pharmacological activation of GABAA receptors or through optogenetic stimulation of inhibitory inputs from the RMTg to the VTA, mitigates stress-induced social avoidance (Ma et al., 2026). Importantly, PPN inputs deliver top-down glutamatergic and cholinergic signals that specifically gate the millisecond-scale phasic transients required for RPE computations (Mena-Segovia et al., 2008). Indeed, pharmacological or excitotoxic lesions of the PPN, as well as local blockade of VTA muscarinic and nicotinic acetylcholine receptors, attenuate or abolish dopamine-mediated RPE signals (Dautan et al., 2016; Xiao et al., 2016). This highlights that local VTA cholinergic receptor balance is critical for dopamine RPE signaling, and that mesopontine sub-regional divergence (e.g., PPN vs. LDT) may differentially shape stress vulnerability versus reinforcement learning mechanics.

### Future direction

Individual variability in stress paradigms far exceeds simple behavioral “susceptible versus resilient” binary dichotomies, highlighting the need for multi-dimensional classification tools that capture behavioral, circuit, and molecular diversity to understand the true spectrum of individual differences in stress neurobiology. Because biological sex fundamentally governs MDD presentation and therapeutic response, treating sex as an essential biological variable is imperative, requiring concrete methodological commitments across all levels of research, including dual-sex inclusion, sex-counterbalanced designs, sex-disaggregated reporting, and sufficiently powered comparative analyses (Finn et al., 2026).

To fully deconstruct the physiological and cellular heterogeneity of DA circuits, future research should leverage modern, genotoxicity-free base editing technologies. CRISPR-Cas9 editings are often used to delineate cellular and projection heterogeneity of DA circuits. Standard CRISPR-Cas9 editing relies on double-strand breaks (DSBs) that frequently trigger extensive genotoxic stress, structural aneuploidy, and p53-dependent toxicity (Zuccaro et al., 2020; Jerabek et al., 2026). In midbrain dopamine (mDA) populations, these DSB-induced alterations can disrupt highly sensitive electrophysiological and metabolic endophenotypes, such as baseline pacemaking activity, mitochondrial respiration, and $I_{h}$ densities. By utilizing DSB-independent base editors delivered as ribonucleoprotein (RNP) complexes (Jerabek et al., 2026), researchers can introduce precise single-nucleotide variants into rodent models without the confounding effects of DNA damage responses, enabling clean manipulation of key developmental and functional loci, such as *Th* and other regulatory regions, axonal guidance cue dynamics during development, and target-specific synaptic connectivity before chronic stress exposure. Crucially, this approach provides a direct translational bridge to human modeling: RNP-based editing enables damage-free genetic modification in human embryonic stem cells (hESCs) and patient-derived induced pluripotent stem cells (iPSCs), which can then be differentiated into mature mDA lineages and 3D midbrain organoids (Jerabek et al., 2026). Combining rodent projection-specific circuit manipulation with human isogenic organoid platforms via precision base editing offers an unprecedented opportunity to resolve the biophysical mechanisms governing stress susceptibility.

Despite intensive efforts to delineate how stressors alter neural circuitry to induce psychiatric phenotypes, the clinical translation of these findings into targeted therapeutics remains constrained. Methodologically accounting for “hidden variables” in preclinical models, specifically variations in behavioral study design, sex and individual differences, and the complex interplay between endocrine stress responses and endogenous circadian or ultradian rhythms (Holloway and Lerner, 2024) is paramount to improve the translational fidelity of stress neurobiology research and unlock novel psychiatric interventions. Discrepancies across preclinical stress paradigms often stem from uncontrolled laboratory variables that skew behavioral and neurophysiological phenotypes. For example, rodents tested during their subjective light phase (inactive period) show baseline corticosterone profiles, dopamine clearance kinetics, and reward sensitivity that diverge sharply from those tested during their dark (active period) phase (Logan et al., 2015). Furthermore, subtle genetic differences between commercial vendor substrains (C57BL/6J vs. C57BL/6N mice) introduce critical phenotypic divides including baseline $I_{h}$ densities and stress susceptibility (Jung et al., 2014). In tandem, animal facility-specific gut microbiome profiles differentially modulate blood-brain barrier permeability, circulating pro-inflammatory cytokines (e.g., IL-6, TNF- α ), and microglial synaptic pruning within the NAc (Cryan et al., 2019), all processes impacted by chronic stress. Finally, individual variability in stress susceptibility is often obscured by categorizing animals into discreate binarized groups (e.g., susceptible vs. resilient), where group averaging often masks meaningful divergence across distinct behavioral and cognitive domains. For example, male mice categorized as resilient based on preserved social interaction following CSDS can still exhibit marked anxiety-like phenotypes (Morel et al., 2022).

Although optogenetic and chemogenetic tools have resolved midbrain circuit causality with remarkable precision, their clinical translation in MDD faces considerable biological and technical barriers. First, these modalities need invasive intracranial hardware and viral delivery vectors (e.g., AAVs) that carry substantial risks of tissue damage, immunogenicity, and uncertain long-term safety (Naldini, 2015). Second, unlike preclinical models, current clinical neuromodulation techniques non-selectively stimulate heterogeneous neuronal populations, glia, and fibers of passage. Third, artificially forcing high-frequency dopaminergic synchronization carries significant psychiatric risks, including the induction of hypomania, psychosis, or addiction. Finally, unlike rodent manipulations, the human brain exhibits extensive homeostatic and network-level compensation across distributed hubs (e.g., default mode and salience networks), which can decrease long-term therapeutic efficacy. To overcome these translational hurdles, future research should pivot toward minimally invasive and closed-loop neuromodulation paradigms such as adaptive deep brain stimulation, focused ultrasound, or temporal interference stimulation, calibrated to real-time electrophysiological signatures of circuit dysfunction. In parallel, developing engineered viral capsids and cell-type-specific synthetic minipromoters could provide projection-defined precision in non-human primates and human clinical trials without requiring germline genomic modification.

While mesocorticolimbic dopamine circuits play a significant role in encoding RPEs, and mediating effort-based motivation and anhedonia, conceptualizing MDD through an exclusively dopamine-centric lens presents distinct clinical and theoretical limitations. MDD is fundamentally a multi-neurotransmitter system disorder involving complex interactions across serotonergic, noradrenergic, cholinergic, glutamatergic, GABAergic, and purinergic networks, alongside large-scale brain network dysregulation (Dunlop and Nemeroff, 2007; Menon, 2011). A primary conceptual challenge in linking dopaminergic RPE mechanics directly to MDD pathophysiology involves a fundamental temporal mismatch: phasic dopaminergic transients operate on a sub-second, millisecond timescale to update immediate reinforcement learning parameters (Schultz, 2025). In contrast, clinical MDD manifests as a chronic, persistent, and slow-evolving state encompassing weeks, months, or years. Thus, blunted phasic RPE signals in depressed individuals likely signify a secondary downstream consequence of chronic network-level stress adaptations, such as sustained cortical E/I imbalance, neuroinflammatory cytokine release, or impaired neuroplasticity, that, overtime, if untreated or unintervened, culminate in a full-blown disorder. This limitation is further underscored by the fact that direct dopaminergic enhancement via L-DOPA, dopamine receptor agonists (e.g., pramipexole), or psychostimulants rarely produces complete or sustained remission in MDD when administered as monotherapy, often failing to resolve core mood disturbances despite transiently elevating arousal or psychomotor activity (Dunlop and Nemeroff, 2007) but also see (Hori and Kunugi, 2012; Costello et al., 2024). Conversely, classical antidepressants (SSRIs, SNRIs) and rapid-acting agents (ketamine) indirectly normalize downstream VTA dopamine population firing by re-establishing prefrontal top-down control or modulating raphe-habenular gating (Belujon and Grace, 2017). Therefore, midbrain dopaminergic modulations or adaptations should be viewed not as a standalone cause of depression, but as an indispensable contributor within an integrated multi-transmitter network.

## Conclusion

Over the past two decades, the neurobiological model of MDD has evolved from a generalized neurochemical deficit into a precise framework of circuit-specific and target-defined microcircuit dysregulation. Chronic stress induces divergent molecular and anatomical adaptations across distinct projection pathways originating from the VTA. As detailed in this review, the pathophysiology of stress-induced dopaminergic dysfunction operates along several distinct biological axes: pathway-specific adaptations (e.g., altered $D_{1}R/D_{2}R$ trafficking in VTA-PFC, and VTA-NAc projections), firing mechanics vs. population activity (e.g., population encoding, neural ensembles), translational trajectory (e.g., individual differences in stress susceptibility, sex-disaggregated paradigms, next-generation editing technologies). Deciphering the exact biophysical, transcriptomic, and circuit-level rules that govern VTA DA heterogeneity remains one of the most promising frontiers in neuroscience and biological psychiatry. By shifting from broad monoaminergic restoration to targeted, circuit-informed therapeutic interventions, future treatments can move closer to precisely reversing the specific neural adaptations that sustain depression-like behaviors.
